# Interventions to improve physical performances of older people with cancer before complex medico-surgical procedures: Protocol for an umbrella review of systematic reviews and meta-analyses

**DOI:** 10.1097/MD.0000000000021780

**Published:** 2020-09-25

**Authors:** Claire Falandry, Laetitia Stefani, Louise Andre, Marion Granger, Claire Barbavara, Hocine Habchi, Chrystelle Bourgeois, Hervé Cure, Guillaume Passot, Thomas Gilbert

**Affiliations:** a Geriatric Unit, Lyon-Sud Hospital; b CarMeN Laboratory, Inserm U1060, INRA U1397, Université Claude Bernard Lyon 1, INSA Lyon, Charles Mérieux Medical School, Lyon University, Oullins; c Oncology Department, Centre Hospitalier Annecy Genevois, Pringy; d Urology Department, University Hospital Jean Monnet, St. Etienne; e Department of Medical Oncology, CHU de Grenoble, La Tronche; f Department of Surgical Oncology, CHU Lyon Sud, Hospices Civils de Lyon, University of Lyon; g Health Services and Performance Research (HESPER EA7425), Lyon, France.

**Keywords:** cancer, older people, prehabilitation, umbrella review

## Abstract

**Background:**

Current demographics lead increasing older cancer patients to undergo complex medico-surgical procedures, with substantial risk of decompensations and deconditioning. The Prehabilitation & Rehabilitation in Oncology: Adaptation to Disease and Accompaniment of Patients’ Trajectories (PROADAPT) project is currently being developed with the aim of improving care, through standardized care pathways guided by existing evidence and implementation programs. A working group will specifically focus on improvement of physical performances before such procedures. These interventions may have been developed in different contexts: before surgery in large, before carcinologic surgery or complex medical interventions (chemotherapy, radiotherapy), or in primary care for elderly patients to prevent sarcopenia and frailty. Post-surgical interventions are out of the scope of this review. The objective of this review is to summarize the level of evidence to support physical reconditioning interventions and identify areas where further work is required.

**Methods:**

This umbrella review will include moderate to high quality systematic reviews, meta-analysis, and pre-existing umbrella or meta-reviews. Two reviewers will independently search the following databases: PubMed/MedLine, Cochrane Library, Embase, and CINAHL. Research strategy will use diverse keywords used to refer to the concepts of “prehabilitation,” “preoperative exercise,” or “preoperative rehabilitation,” with prespecified inclusion and exclusion criteria and only systematic reviews selection. The distinct types of interventions presented using PRISMA guidelines and a narrative reporting of results. A focus will be made on outcomes such as physical performances, quality of life, autonomy in everyday activities, or number of hospital bed days.

**Ethics and dissemination:**

Ethical approval is not required for such an umbrella review. Our review will be submitted for publication in a peer-reviewed international journal using open access option if available. It will be complementary to reviews focused on hospital discharge of older people.

**PROSPERO registration number:**

CRD42020100110.

## 1 Introduction

In 2016, cancer has become in Western Europe the first cause of death in patients over 65, a global trend due to the decrease in cardiovascular mortality and an increasing incidence of cancer with age.^[1]^ Moreover, cancer itself and cancer treatments are a major cause of disability and physical deconditioning in the older population. Recent data from the Surveillance Epidemiology and End Results Program (SEER) demonstrated that, apart from direct surgical outcomes, usually evaluated using Clavien-Dindo classification of post-surgical adverse events, older people who underwent major carcinologic surgeries display a substantial risk of hospital admissions in the 30 days after surgery for geriatric events.^[2]^ Consequently, treatment decisions may have a major impact, both medical and economical, both immediate on patients and the health system through patients’ care-courses and adverse events management, and in the long term on patients and their families through their long-term impact on functionality and quality of life. The health systems of developed countries face the dual issue of aging demographics and budget restriction, which push towards an increased turn-over of hospital beds and reduced length of stay. In this context, there is a need to develop intervention programs to decrease the risk of functional deconditioning in this high-risk population.

Prehabilitation has been for long conceptualized as an effective way to enhance functional capacity of the individual to enable him or her to withstand different stressors. Initially developed in the army as the association of physical training to improve strength and endurance, improved nutritional intake and general education, it was transposed to medicine and major surgery—particularly elective cardiac and orthopedic surgery—at the beginning of this century. The concept of cancer prehabilitation arose from that context as “a process on the cancer continuum of care that occurs between the time of cancer diagnosis and the beginning of acute treatment and includes physical and psychological assessments that establish a baseline functional level, identify impairments, and provide interventions that promote physical and psychological health to reduce the incidence and/or severity of future impairments.”

Despite an increasing interest of medical community on prehabilitation and particularly cancer prehabilitation, the level of evidence for specific interventions is considered as too low to be implemented in common care. Among the major drawbacks of published data are the heterogeneity of the programs, sometimes a poor adherence of the patients and the fact that most studies were small pilot studies that were developed for fitter and younger patients than those expected to get the better benefit from prehabilitation. Another point to highlight is the fact that the majority of prehabilitation programs include only one intervention—either a physical, nutritional, or psychological prehabilitation—when multimodal interventions are frequently considered as more effective in older populations. Nevertheless, 71% of the surgeons interviewed would accept to prehabilitate their elderly patients 4 weeks before surgery, if such intervention is proven to be effective.^[3]^

Moreover, as the older population appears particularly vulnerable, with an increased risk of adverse outcomes and unplanned readmissions after discharge,^[4]^ as his medico-social management is more complex and the risks are higher, the old patient is considered as a good target to demonstrate the medico-economic impact of such prehabilitation strategies. In the context of cancer care, older patients appear even more at risk, as the burden of the disease and treatments add up to the patient's underlying condition. Even the fittest older patients may be strongly affected by complex treatments such as chemotherapy, radiotherapy, or heavy surgical procedures, hence widening the scope of frailty.^[5]^

**Figure 1:**
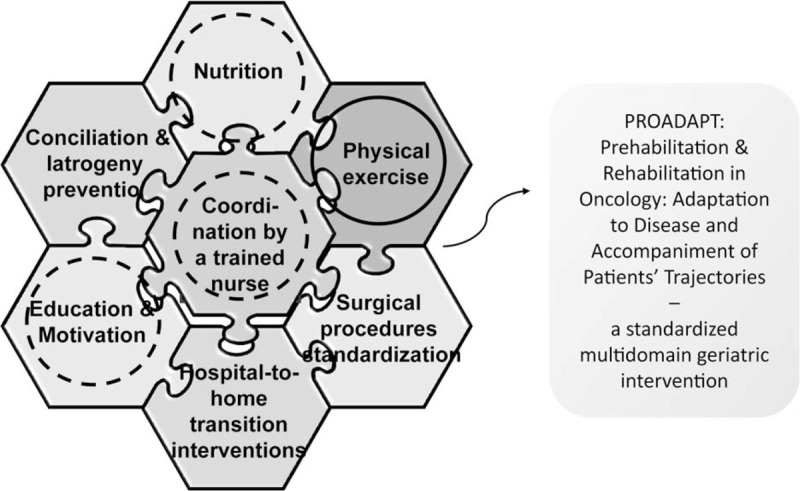
The global design of PROADAPT standardized multidomain geriatric intervention (dashed lines: contents included in the current meta-review on physical exercise prehabilitation; dotted lines: contents possibly associated to physical exercise interventions in the current meta-review). PROADAPT = prehabilitation & rehabilitation in oncology: adaptation to disease and accompaniment of patients.

The PROADAPT project is currently being developed to address this concern, with the aim of improving outcomes of older patients undertaking complex medical and surgical treatments for cancer, through harmonized evidence-based pathways of care and procedures. In a holistic approach, various aspects of care and prevention will be covered by this program (nutritional status, pre/rehabilitation, standardization of procedures, medical reconciliation, patient education, and transition) (Fig. 1).

## 2 Objectives

The overarching aim of this review is to summarize and perform a grading of the level evidence in the literature on prehabilitation interventions (physical ± nutritional ± psychological) that may apply to older patients who will undergo complex medico-surgical procedures for cancer treatment. We will aim to answer the following questions:

- Which interventions are efficient?
- For which patients/in which context?
- For which outcomes?
- And if possible, how are they best implemented?

Due to the paucity of published results in the specific field of the research topic, this umbrella review will aim at covering in large interventions designed for adult patients before surgery or complex medical interventions, either in the context of cancer or in other settings.

## 3 Methods and analysis

### 3.1 Criteria for included studies

#### 3.1.1 Type of study

We will focus the literature search on existing high quality systematic reviews, meta-analysis and systematic mapping reviews. We will follow the PRISMA guidelines for conducting this review.^[6]^ The included reviews will need to:

- Clearly state the research question and the objectives of the study.
- Include an explicit methods section with the eligibility criteria for included studies, a reproducible methodology with a systematic approach that attempts to identify all studies that would meet the eligibility criteria.
- Show a predefined quality appraisal of included studies with validated methodology.
- Describe a systematic method for data extraction, synthesis and presentation of findings.

Existing guidelines, expert consensus papers, randomized controlled trials performed and reported more recently than those reported in the included reviews, and existing qualitative studies focused on implementation of prehabilitation programs will not be included in the umbrella review, but might be considered as complementary information while analyzing the results.

#### 3.1.2 Population and context

The overarching aim of this umbrella review is to gather evidence on prehabilitation of older patients planned to undergo complex treatments for cancer. However, programs designed towards broader age groups or in other contexts than cancer may still be relevant and possibly transposable to this specific purpose.

Therefore, we will include reviews of prehabilitation interventions designed for:

- Older patients (>65 years) planned to undergo major oncologic surgery and/or complex medical interventions as chemotherapy, radiotherapy.
- Older patients (>65 years) planned to undergo non-oncologic major surgery (elective cardiac, orthopedic, digestive…).
- Older patients (>65 years) in primary care.
- Patients (not specifically old) planned to undergo major oncologic surgery and/or complex medical interventions as chemotherapy, radiotherapy.
- Patients (not specifically old) planned to undergo non-oncologic major surgery (elective cardiac, orthopedic, digestive…).

Five meta-reviews are currently underway and identified in PROSPERO as “The effectiveness of prehabilitation for adults having elective surgery: a systematic review protocol,^[7]^” “The effectiveness of prehabilitation on long term outcome measures for adults due to undergo treatment for cancer: a systematic review protocol,^[8]^” “Prehabilitation programmes for newly diagnosed cancer patients,^[9]^” “Multimodal prehabilitation programs as a bundle of care in gastrointestinal cancer surgery: systematic review^[10]^” and “A systematic review and meta-analysis of nutrition prehabilitation with or without exercise^[11]^” that will enrich our conclusions as soon as they are published before the end of data extraction. These works will be complementary to ours and we will take the results of this review into account according this umbrella review methodology rather than duplicating the review process.

#### 3.1.3 Interventions and comparators

All interventions specifically designed to improve physical performance (strength and endurance) ± nutrition ± psychology and adherence will be considered (sometimes, these interventions will be associated within multi-modal programs but physical exercise is mandatory). This can include (not exclusively):

##### 3.1.3.1 Various types of exercises

- Aerobic (=endurance) training: cycling, walking, aquatic exercises…
- Resistance training (=strengthening).
- Pulmonary exercises (=breathing): triflow,
- Abdominal exercises,
- Pelvic exercises (=continence training).

##### 3.1.3.2 Various modalities of supervision

- Supervised exercise ± biofeedback.
- Unsupervised exercise.
- Group activities.
- Written instructions.
- Verbal instructions.
- Current guidelines prescription.

Various durations of the prehabilitation program.

Various duration of each training session.

Various frequencies of training sessions.

Various intensities and intensity scales, number of repetitions for strength exercises: steps, contractions…

#### 3.1.4 Outcomes

The quality of prehabilitation can be evaluated on many different outcomes from the patient and the system's point of view (e.g., VO2 peak; functionality, urinary or rectum continence, mortality, postoperative complications, patient satisfaction, quality of life, length of stay, costs, ...). These outcomes will be listed and mapped during the review process.

For example, many interventions have been designed to decrease length of stay.^[12,13]^ Even though such interventions may also have beneficial effects on patient-related outcomes, vulnerable older patients treated for cancer may still have an important risk of unplanned readmissions.

Another dimension to be highlighted in a specific population of older adults is the acceptability of the intervention program. Bruns recently highlighted in a systemic review that the compliance to preoperative exercise varied from 16% to 97% in elderly patients undergoing colorectal surgery.^[14]^ As this meta-review focuses on prehabilitation programs to be proposed to a heterogeneous population, eventually frail and psychologically distressed, a particular attention will be paid to compliance data.

#### 3.1.5 Exclusion criteria

- Interventions in the paediatric context.
- Interventions in the context of mental health care or psychiatry, except from delirium or dementia-orientated services.
- Palliative support. Even if oncology patients may be likely to deteriorate, the aim of the PROADAPT program is to define methods for preventive interventions in actively treated patients rather than promoting palliative care, which is already well structured in this context.
- Interventions not designed as prehabilitation exercise programs.
- Reviews for which the full report is not available (e.g., published protocol with no final report available).
- Reviews written in other languages than English or French (for pragmatic reasons), which will still be considered up to the English language version of the abstract during the selection process.
- Reviews with insufficient quality based on the AMSTAR-2 check-list measurement tool to assess systematic reviews.^[15]^

### 3.2 Study retrieval

#### 3.2.1 Sources

- Database searches: PubMed/MedLine, Cochrane Library, Embase, and CINAHL.
- Ongoing studies: PROSPERO.
- Grey literature: As this umbrella review will include systematic reviews of moderate to high quality, we will assume that they have sought grey literature and that they will have been published in indexed journals.

Limits: the initial search will be limited to the 2000 to 2020 period, as we will be searching for up-to-date information. However, this period might be extended if there are too few results to the search.

#### 3.2.2 Timeline

The search will be conducted from January 2020 and repeated monthly up to December 2020. When possible, alerts will also be set in main databases such as PubMed.

#### 3.2.3 Search strategy

In accordance with the scope of the research, three main concepts can be drawn out of the research question:

- Older patients
- Physical prehabilitation
- Cancer

**Figure 2:**
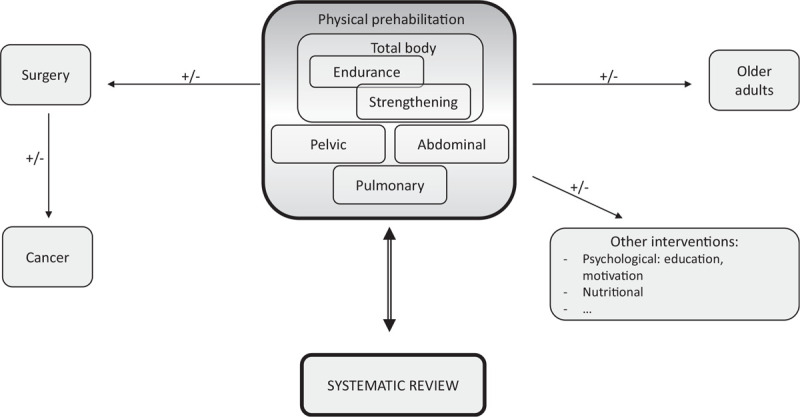
Simplified outline of the search strategy.

The outline of the search strategy is pictured in Fig. 2.

#### 3.2.4 Search terms

Searches will be developed and combined using broad search terms, key words and MeSH terms referring to each of the concepts pictured above (this is list is not comprehensive and subject to evolution):

- Older adults (e.g., old∗, aged, aging, elder∗, senior∗, geriatric∗, frail, late-life, pensioner∗, veteran∗)
- Prehabilitation, preoperative rehabilitation, preoperative exercise, etc.
- Cancer, cancer treatment, neoplasms, tumour, surgical recovery
- Review, Systematic review, meta-analysis, meta-review, mapping review

#### 3.2.5 Detailed search strategy

The search strategies are presented in Table 1 according different databases.

**Table 1:**
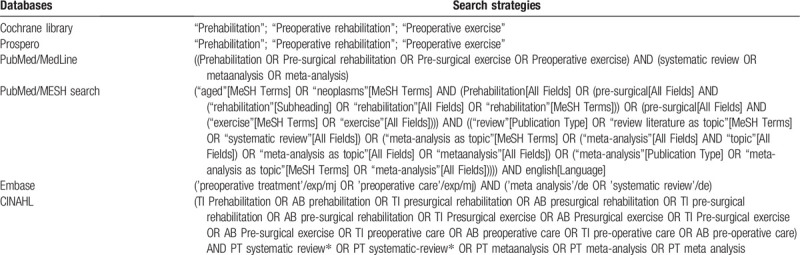
Search strategies.

#### 3.2.6 Study selection

The number of results for each database search and with other sources will be noted and presented in a PRISMA flow-diagram, which will be updated and finalised in December 2020.

##### 3.2.6.1 Title and abstract selection

First, all the citations will be imported in Zotero and duplicates will be removed. After title screening, all potentially relevant citations will be exported to Microsoft Excel with full title, author, journal and year.

Two authors will then independently screen titles and abstracts for inclusion and exclusion criteria. Each decision to include or exclude a study will be marked by 1 or 0 by both reviewers to allow the evaluation of inter-rater agreement between reviewers. The inclusion scores between the two reviewers will be then be added up in Excel, and lead to three possible scores: 2 (selection for full-text review), 1 (need for discussion between authors), and 0 (exclusion). Two Excel files will initially be completed independently by two authors, and will then be merged into one and shared on a Google drive. Duplicates will be removed during the merging process.

All articles selected using this method will be considered for the full-text selection stage.

A Venn diagram will be constructed in order to evaluate the level of redoundancy between the different databases.

##### 3.2.6.2 Full-text selection

After the initial title and abstract selection, the full-text articles will be obtained and screened independently for inclusion and exclusion criteria by 2 reviewers. This process will comprise the quality assessment of the reviews (see quality assessment).

Similarly to the previous step, each decision to include or exclude a study will be marked by 1 or 0 by both reviewers. This will allow the evaluation of inter-rater agreement between reviewers and unable to create a global score of 0 (exclusion), 1 (unclear, discussion), or 2 (inclusion).

At this stage, all exclusion decisions will be documented and recorded to allow the results of the screening to be reviewed. AMSTAR-2 scores will also be reported independently by both authors.

##### 3.2.6.3 Follow-up of bibliography of key articles

After the full-text selection, the bibliography of all articles included by both authors will be screened in search for possible additional references, for which the process would be repeated from the title and abstract selection stage.

##### 3.2.6.4 Critical appraisal

For the critical appraisal of the studies, we will apply the AMSTAR-2 checklist to select only the reviews of high to moderate quality, defined using the AMSTAR-2 check-list.^[15]^ In accordance with previous or ongoing studies using AMSTAR-2 for quality assessment of systematic reviews, the studies will be marked as of high quality if they present 0 to 1 non-critical weakness; of moderate quality if they have >1 non-critical weakness; of low quality if they present one critical flaw with or without non-critical weaknesses: of critically low quality if they have >1 critical flaw with or without non-critical weaknesses.^[15–17]^

### 3.3 Data analysis plan

A “flow diagram” charting the number of references at each stage in the review process will be produced in line with the PRISMA statement.^[6]^ The quantity and quality of the literature will be summarized in both narrative commentary and summary tables, which may be adapted according to the results of the umbrella review.

The articles selected for full-text review will be screened according to a standard Data extraction form.

A full report will be developed, which will include a narrative overview with detailed description of the review methodology and findings.

The initial draft of manuscript will be circulated within the research team and coinvestigators among the PROADAPT group. Depending on the results of this review, we may be able to present a set of guidelines for good practice in accordance with the available evidence. For grading of recommendations, we will then use the GRADE guidance.^[18]^

The manuscript will be amended until a consensus is reached with regards to the recommendations which can be drawn out of the umbrella review.

## 4 Ethics and dissemination

This review will aim to be as comprehensive as possible. In line with the concept of umbrella reviews or meta-reviews, sources of grey literature will not be sought directly. However, this umbrella review will include only systematic reviews, meta-analysis or meta-reviews of moderate to high quality, which should thus have considered grey literature. This should help to reduce the risk of publication bias. However, the exclusion of reviews in other languages than English or French may reduce the scope of the research.

This systematic umbrella review should help us to evaluate the acceptability and efficiency of different physical exercise programs with the level of evidence to support their implementation in the PROADAPT program. However, it is only focused on high quality systematic reviews and it is likely that it will leave many gaps due to insufficient quality evidence. The first steps of our analyse demonstrated that the manuscripts included in the different meta-analyses already published are not purely redundant, leading to potential misinterpretations. We expect that including the whole content of these systematic analyses will improve the interpretation. We are aware that some questions relevant to our work might be left unanswered. It may, thus, also be useful to widen the scope to reviews of lower quality evidence or expert consensus publications. Such sources could not be included in this review, which aims for high quality information in priority, but might be considered as complementary information while compiling the results of the review. Another conflicting point is located in the high heterogeneity of the interventions tested has been highlighted in many previous articles, leading to systematic reviews instead of real meta-analyses.^[19]^ The research team will also take into account existing guidelines from health authorities or expert groups. Nevertheless, this review will help to orientate future dedicated systematic reviews of randomised trials or new research projects on the topic, so that as much reliable information as possible can be gathered.

This study will focus specifically on older patients with cancer. We anticipate that this refined population may lead to few results. However, we felt that given the increasing numbers of older patients being treated for cancer, a systematic approach towards important level evidence would still be useful, even if this review only helps to draw out priorities for future (needed) research.

During the next step, while designing the implementation program as part of the PROADAPT project, we will use a Delphi method with a multi-professional group of stakeholders from different settings.^[20]^ This should help to address this concern. Our review may also provide a description of settings and implementation of the programs as reported in the included studies. Yet, a complementary review of qualitative studies with a realistic approach, may be deemed necessary to better understand the key elements and barriers for implementation of transition and patient education programs.

## Acknowledgments

This review project is part of the PROADAPT initiative for improving care for older people with cancer. The authors wish to thank the professionals who participated to the group analyses: G. Albrand, D. Barnoud, D. Benayoun. B Billod, J. Bonhomme, A.-L. Bres, C. Brunengo, E. Castel-Kremer, D. Charlety, Y. Chauleur, G. Copaescu, A.-M. Dascalita, B. De La Vigerie, S. Ducoulombier, B. Galamand, E. Genest, M. Giroud, M. Haine, N. Jomard, C. Lecardonnel, B. Leroy, J.-A. Long, A. Marion, I. Morel-Soldner, E. Nony, A. Pelisset-Vanhersecke, A. Pirollet, C. Ravot, J.-E. Terrier, J. Trautmann, A.-C. Vincent.

## Author contributions

**Conceptualization:** Claire Falandry, Laetitia Stefani, Marion Granger, Claire Barbavara, Hocine Habchi, Chrystelle Bourgeois, Hervé Cure, Guillaume Passot, Thomas Gilbert.

**Data curation:** Claire Falandry, Marion Granger, Claire Barbavara, Hocine Habchi, Chrystelle Bourgeois, Guillaume Passot, Thomas Gilbert.

**Formal analysis:** Claire Falandry, Louise Andre.

**Funding acquisition:** Claire Falandry.

**Investigation:** Claire Falandry, Louise Andre.

**Methodology:** Claire Falandry, Louise Andre.

**Project administration:** Claire Falandry.

**Resources:** Claire Falandry.

**Software:** Claire Falandry.

**Supervision:** Claire Falandry, Thomas Gilbert.

**Validation:** Claire Falandry, Laetitia Stefani, Louise Andre, Marion Granger, Claire Barbavara, Hocine Habchi.

**Visualization:** Claire Falandry, Laetitia Stefani, Louise Andre, Marion Granger, Claire Barbavara, Hocine Habchi, Chrystelle Bourgeois, Hervé Cure, Guillaume Passot, Thomas Gilbert.

**Writing – original draft:** Claire Falandry, Thomas Gilbert.

**Writing – review & editing:** Claire Falandry, Laetitia Stefani, Louise Andre, Marion Granger, Claire Barbavara, Hocine Habchi, Chrystelle Bourgeois, Hervé Cure, Guillaume Passot, Thomas Gilbert.
